# Регистры заболеваний околощитовидных желез в Российской Федерации

**DOI:** 10.14341/probl12803

**Published:** 2021-08-19

**Authors:** Н. Г. Мокрышева, Е. В. Ковалева, А. К. Еремкина

**Affiliations:** Национальный медицинский исследовательский центр эндокринологии; Национальный медицинский исследовательский центр эндокринологии; Национальный медицинский исследовательский центр эндокринологии

**Keywords:** первичный гиперпаратиреоз, гипопаратиреоз, регистры, паратгормон

## Abstract

На сегодняшний день наиболее важным и рациональным способом организации эндокринной службы в масштабах страны, осуществляющей преемственность, маршрутизацию, а также расчет необходимых объемов оказания медицинской помощи, представляется создание единой карты ведения эндокринного больного (Эндокарты).Необходимыми этапами создания единой Эндокарты являются информационно-аналитические платформы регистров эндокринопатий, уже сейчас поставляющие информацию об основных эпидемиологических и клинических характеристиках социально значимых заболеваний, таких как сахарный диабет.Учитывая отсутствие широкомасштабных эпидемиологических данных по проблемам заболеваний околощитовидных желез — первичного гиперпаратиреоза и гипопаратиреоза, внедрение и ведение регистров по этим нозологиям с возможностью удаленного доступа к информации по регионам, безусловно, обладает высоким уровнем практической новизны и научного потенциала.

## Abstract (en)

The most important and effective way to organize nationwide the healthcare, as well as monitoring and routing for patients with endocrine diseases, is the creation of an unified medical record (Endocard). The Endocard is also aimed at maximizing the opportunity for professionals and researchers on various scientific issues. Registries are the potential informational and analytical platform to achieve this goal. They include the basic information on the epidemiological and clinical features of the most severe diseases such as diabetes mellitus. Given the lack of large-scale epidemiological data on the parathyroid glands pathology — primary hyperparathyroidism and hypoparathyroidism — the registers of these diseases that collects a common dataset and clinician and patient reported outcomes are of particular interest.

## ВВЕДЕНИЕ

Околощитовидные железы (ОЩЖ) — железы эндокринной системы, являющиеся важнейшими регуляторами минерального обмена. Поддержание стабильного уровня кальция крови происходит за счет продукции и секреции паратиреоидного гормона (ПТГ) ОЩЖ. В условиях его избытка, при опухолевой трансформации ОЩЖ развивается первичный гиперпаратиреоз, при его недостатке — гипопаратиреоз. Оба состояния не только напрямую воздействуют на фосфорно-кальциевый обмен, но и оказывают влияние на многие другие органы и системы, в частности сердечно-сосудистую систему, почки и костную ткань.

Практика создания и ведения регистров пациентов в последнее время становится все более актуальной в медицинском сообществе. Интерес к регистрации пациентов с различными нозологиями обусловлен потребностью в адекватной и точной информации о клиническом течении заболевания, эффективности применяемых медицинских мероприятий, мерах профилактики в условиях реальной клинической практики. Использование регистра в системе здравоохранения государства является удобным и доступным инструментом для решения большого количества организационных и исследовательских задач.

## ПЕРВИЧНЫЙ ГИПЕРПАРАТИРЕОЗ

Первичный гиперпаратиреоз (ПГПТ) — заболевание, характеризующееся гиперсекрецией ПТГ вследствие первичного поражения ОЩЖ и проявляющееся нарушением фосфорно-кальциевого обмена с вовлечением в патологический процесс различных органов и систем.

Спорадическим ПГПТ является в 90–95% случаев, около 5–10% случаев ПГПТ составляют наследственные формы и проявляются либо как изолированное заболевание, либо в сочетании с другими клиническими проявлениями [1][2]. ПГПТ, сопровождающийся гиперплазией нескольких ОЩЖ или множественными аденомами, как правило, сочетается с наследственными синдромами: множественными эндокринными неоплазиями типа (МЭН)-1, МЭН-2, МЭН-4, синдромом гиперпаратиреоза с опухолью нижней челюсти (hyperparathyroidism-jaw tumour syndrome, HPT-JT), семейным изолированным гиперпаратиреозом и семейной гипокальциурической гиперкальциемией (familial hypocalciuric hypercalcemia, FHH) [3–6].

ПГПТ ассоциирован с поражением многих систем организма, с повышением смертности, в том числе из-за снижения функции почек, развития остеопороза с риском низкотравматических переломов. По данным ряда исследований, у пациентов с ПГПТ существует более высокий риск развития сердечно-сосудистых заболеваний (ССЗ), что также приводит к повышению смертности от них по сравнению с общей популяцией. Большинство работ указывает на то, что повышенный уровень сывороточного кальция является независимым предиктором смерти от ССЗ [7][8].

ПГПТ относится к одним из наиболее распространенных эндокринопатий, занимая третье место после сахарного диабета и заболеваний щитовидной железы по данным зарубежных источников. В общей популяции распространенность ПГПТ составляет в среднем около 0,86–1% [9]. За последние десятилетия отмечено резкое увеличение заболеваемости ПГПТ, в большей степени за счет выявления бессимптомных форм заболевания, не сопровождающихся высокой гиперкальциемией и ассоциированных с ней осложнений.

Для оценки выявляемости ПГПТ и его эпидемиологической составляющей, а также характеристики его форм в Российской Федерации (РФ) был создан Всероссийский онлайн-регистр ПГПТ. В настоящее время в базе данных (онлайн-регистре) содержатся данные более чем о 4400 пациентах из 79 регионов РФ (рис. 1).

**Figure:**
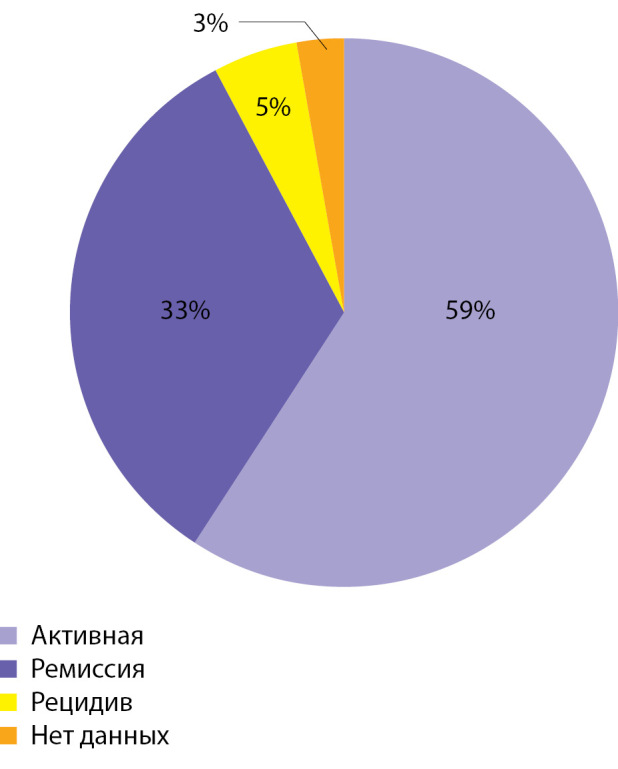
Рисунок 1. Текущее распределение пациентов с ПГПТ по фазе течения заболевания.

Средний возраст больных на момент постановки диагноза составляет 56,7±13 лет, что согласуется с результатами зарубежных исследований. Отмечается значимое превалирование заболевания среди женщин. Активная фаза заболевания на момент представления данных зарегистрирована у 59% пациентов, при этом у большинства (58,8%) верифицированы ПГПТ-ассоциированные осложнения со стороны почек и костей. В случае симптомной формы по сравнению с бессимптомным течением ПГПТ выявляются достоверно более высокие уровни ПТГ и кальция в сыворотке крови, таким образом они могут рассматриваться как некие индикаторы тяжести течения заболевания на момент первичной постановки диагноза (p<0,05).

Особое внимание в рамках работы регистра уделяется наследственным формам ПГПТ, в том числе в составе МЭН, и злокачественному поражению околощитовидных желез. Клиническим критериям синдромов МЭН-1 и МЭН-2А соответствуют 669 пациентов, средний возраст 43,62±15,9 года. Генетическое тестирование проведено 114 человек (2,7%). Среди них мутации в гене MEN1 зафиксированы в 1,7% случаев (71/4176) и в гене CDC73 — в 0,14% случаев (6/4176). 7 пациентов имеют сочетание ПГПТ и медуллярного рака щитовидной железы. Результаты послеоперационного морфологического исследования свидетельствуют о достаточно высокой заболеваемости раком ОЩЖ, которая составила 1,9%.

Дальнейший углубленный комплексный фармако-эпидемиологический и клинико-экономический анализ регистра ПГПТ, проведенный в условиях реальной клинической практики, выступит инструментом в принятии решений при планировании расходов государства на оказание первичной, специализированной и высокотехнологичной медицинской помощи. В настоящее время в России прослеживается увеличение частоты выявления мягкой формы ПГПТ [10]. Однако отсутствие четких критериев ее диагностики приводит к различиям по рекомендуемой тактике ведения пациентов и подчеркивает необходимость дальнейшего изучения распространенности ПГПТ.

## ГИПОПАРАТИРЕОЗ

Гипопаратиреоз — состояние, характеризующееся сниженной продукцией ПТГ ОЩЖ или резистентностью тканей к его действию, что сопровождается нарушениями фосфорно-кальциевого обмена.

Самой частой этиологией заболевания является развитие послеоперационного гипопаратиреоза (до 75% всех случаев) после хирургического лечения заболеваний органов шеи, как правило, щитовидной железы. Радикальное лечение ПГПТ также может стать причиной развития гипопаратиреоза, особенно в случаях первично множественного поражении ОЩЖ, например, при синдромах МЭН. К более редким случаям, выявляемым в основном в детском возрасте, относят аутоиммунный гипопаратиреоз, аутосомно-доминантную гипокальциемию и другие наследственные формы заболевания [11][12].

Развитие хронического гипопаратиреоза любой этиологии требует пожизненного назначения многокомпонентной терапии, а также тщательного мониторинга и индивидуального подхода к ведению заболевания. Ввиду отсутствия патогенетического лечения гипопаратиреоза в настоящее время проводится симптоматическое, требующее четкого контроля. При отсутствии адекватного динамического наблюдения развиваются множественные осложнения со стороны жизненно важных органов, в частности патология почек и ССЗ, зрительные нарушения, развитие нейрокогнитивных расстройств, что приводит к резкому снижению качества жизни [13].

Гипопаратиреоз, по данным зарубежной литературы, является достаточно редким заболеванием с распространенностью 23–46 на 100 тыс. населения [14], однако распространенность на территории РФ в настоящее время неизвестна.

Опыт ведения единой национальной базы данных, представленный в Дании и некоторых других странах Европейского союза, показывает ценность централизации и единой систематизации информации, которая позволяет оценивать как эпидемиологические характеристики хронического гипопаратиреоза с возможностями прогнозирования роста заболеваемости или инвалидизации пациентов в связи с развитием терминальных осложнений, выявлять риски развития данного заболевания и, по возможности, их корректировать, так и оценивать качество оказания медицинской помощи, в том числе в различных областях с учетом локальных особенностей.

В РФ создание всероссийской электронной платформы (онлайн-регистра) пациентов с хроническим послеоперационным и нехирургическим гипопаратиреозом произошло сравнительно недавно, в конце 2020 г. На текущий момент регистр включает информацию о более чем 570 пациентах с данным редким заболеванием, и эта цифра постоянно растет по мере включения новых регионов в работу регистра (рис. 2).

**Figure:**
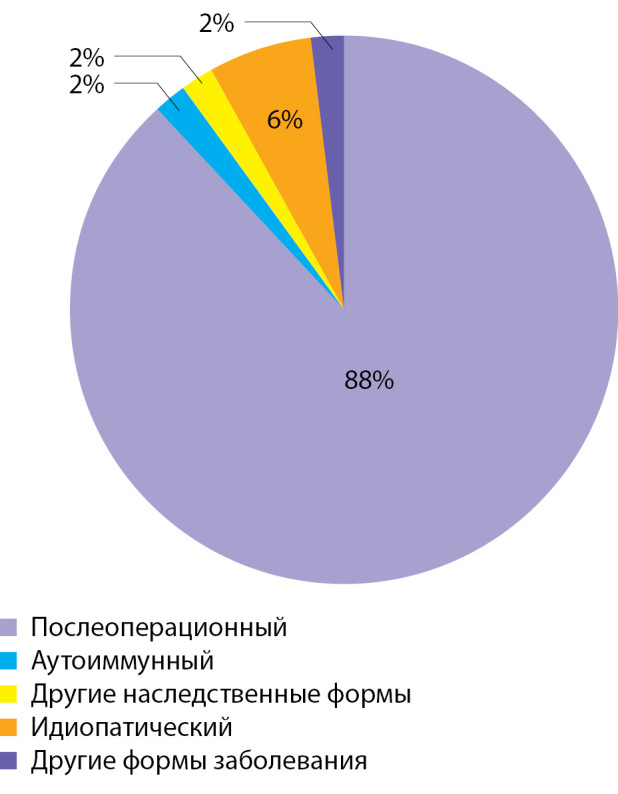
Рисунок 2. Распределение пациентов с гипопаратиреозом по этиологии заболевания.

По результатам анализа базы данных пациентов с гипопаратиреозом ФГБУ «НМИЦ эндокринологии» Минздрава России, составившей основу регистра гипопаратиреоза, было установлено, что менее половины пациентов имеют целевые показатели кальция и фосфора сыворотки крови (31 и 47% соответственно), что подтверждает отсутствие компенсации заболевания у большинства больных и требует оптимизации медикаментозного лечения и наблюдения. Второй проблемой стало понимание недостаточности обследования пациентов — только в 58% случаев проводился необходимый комплекс инструментальных диагностических мероприятий. Внедренная в онлайн-регистр гипопаратиреоза система поддержки принятия решений призвана улучшить качество диагностики и лечения данных пациентов, в том числе путем выведения информационных подсказок о необходимости дообследования по ключевым параметрам фосфорно-кальциевого обмена, изложенным в утвержденных в 2021г. клинических рекомендациях по гипопаратиреозу [15].

## ЗАКЛЮЧЕНИЕ

На сегодняшний день к одной из приоритетных задач здравоохранения относится создание единой системы учета эндокринных заболеваний — эндокринного паспорта пациента. Именно такой подход позволяет унифицировать клинико-эпидемиологический мониторинг в целом и персонализированно для конкретного пациента с целью оценки распространенности, заболеваемости, характера течения, исходов, оптимальных алгоритмов лечения и профилактики эндокринопатий в РФ. Необходимыми шагами в достижении этой цели является создание Всероссийских баз данных, в которых аккумулируется вся необходимая информация.
